# Effect of a microencapsulated phyto/phycogenic blend supplementation on growth performance, processing parameters, meat quality, and sensory profile in male broilers

**DOI:** 10.3389/fvets.2024.1382535

**Published:** 2024-03-28

**Authors:** Garrett J. Mullenix, Elizabeth S. Greene, Alison Ramser, Clay Maynard, Sami Dridi

**Affiliations:** Department of Poultry Science, University of Arkansas, Fayetteville, AR, United States

**Keywords:** feed additive, NUQO-NEX, body weight, feed intake, meat color

## Abstract

Powered by consumer taste, value, and preferences, natural products including phytogenics and algae are increasingly and separately used in the food systems where they have been reported to improve growth performance in poultry and livestock. The present study aimed to determine the effects of a new feed additive, microencapsulated NUQO© NEX, which contains a combination of phytogenic and phycogenic, on broiler growth performance, blood chemistry, bone health, meat quality and sensory profile. Male Cobb500 chicks (*n* = 1,197) were fed a 3-phase feeding intervals; 1–14d starter, 15–28d grower, and 29–40d finisher. The dietary treatments included a corn-soy basal Control (CON), basal diet supplemented with NUQO© NEX at 100 g/ton from 1 to 28d then 75 g/ton from d 28 to 40 (NEX75), and basal diet supplemented with NUQO© NEX at 100 g/ton from 1 to 40d (NEX100). The NEX100 supplemented birds had 62 g more BWG increase and 2.1-point improvement in FCR compared with CON in the finisher and overall growth phase (*p* < 0.05), respectively. Day 40 processing body weights and carcass weights were heavier for the NEX100 supplemented birds (*p* < 0.05). The incidences of muscle myopathies were also higher in NEX treatments, which could be associated with the heavier weights, but the differences were not detected to be significant. The NEX75 breast filets had more yellowness than other dietary treatments (*p* = 0.003) and the NEX 100 treatment reduced the levels of breast filet TBARS at 7 days-post harvest (*p* = 0.053). Finally, both NEX treatments reduced the incidence of severe bone (tibia and femur) lesions. In conclusion, the supplementation of the phytogenic NUQO© NEX improved finisher performance parameters, whole phase FCR, processing carcass weights, and breast filet yellowness, at varying inclusion levels.

## Introduction

The continuous increase in demand for animal-source protein is anticipated to escalate globally to approximately 152 Megatonne by 2030, and poultry meat is expected to account for a majority (52%) of the projected growth (1). This anticipated increase in poultry production will require alternatives to classical growth and health promotors which meet government regulations and consumer preferences and demand.

Antibiotics have been extensively used as health mediators and growth promotors (AGP) in animal production for over 70 years, but the increased concerns over antimicrobial resistance have led numerous countries to implement legislation to ban or reduce their usage (2). Sweden was the first European country to prohibit antimicrobials in feedstuffs in 1986; a move that set the groundwork for the European Union implementing an antimicrobial monitoring system (ESVAC) in 2005 and eliminating their use as growth promotors in 2006 (3). There have been efforts in the United States to curtail the usage of antimicrobials in animal production dating back to the 1950s, yet there has been less of a proclivity to truly implement the regulations (4, 5). The USDA banned the usage of antibiotics as growth promotors in 2017, which triggered a shift toward their usage as health promotors with identical or nearly identical dosages (5). The term “no antibiotic ever-NAE” or “raised without antibiotics, RWA” are consumer driven and producer implemented guidelines that have rose to prominence in response to consumers wanting a perceivably more natural protein option.

The poultry industry has been successful in reducing its dependency on antimicrobials, due in large part to its implementation of growth promoting alternatives. Numerous strategies to maintain performance have been used, including probiotics, prebiotics, synbiotics, organic acids, enzymes, antimicrobial peptides, hyperimmune egg antibodies, bacteriophages, clay, metals, algae, and phytogenics (6–9). While these alternatives provide tremendous potential to improve the overall health and performance of the bird, there is no panacea to meet at all the needs of a commercial producer (10). Plant extracts or phytogenics are of exceptional interest because they have been shown to display some beneficial effects observed with AGP, while coming from natural sources (6, 11, 12). Phytogenics are plant-derived compounds that are further classified into herbs (flowering nonpersistent plants), spices (herbs with pungent smell and associated taste), essential oils (volatile lipophilic compounds), or oleoresins (extracts from nonaqueous solutions) (13). The bioactivity of the phytogenics can vary widely between products and can depend on the part of plant used, growing conditions, harvesting conditions, processing/extraction process, and storage conditions (13). A large number of botanical options include bioactive compounds such as polyphenols (flavonoids, stilbenes, lignans, and phenolic acids), phenolics (tannins), glycosides, and alkaloids (14–19) provide beneficial effects in chickens through improved growth performance, digestibility, microbiota, immune response, oxidant status, egg quality, and meat quality (20). A growing number of phytogenics options, such as cinnamon, cloves, coriander, cumin, garlic, ginger, green tea, marjoram, mint, oregano, rosemary, sage, thyme, and yarrow have been tested in avian species, with varying levels of success (20). Similarly, algae extracts and compounds (phycogenics) have tremendous potential as animal feed due to the presence of essential biomolecules such as amino acids, poly-unsaturated fatty acids, carotenoids, and vitamins (21). Increasing number of researches have shown that blending feed with algae can positively affect the growth, health, and overall animal physiology and products at quantitative and qualitative levels (22–27).

Although the abovementioned feed additive alternatives were used extensively with evident beneficial effects, the dietary usage of a combined blend of phytogenics and phycogenics are very limited. Recently, using a double micro-encapsulation technology, a new feed additive (NUQO©-NEX, NUQO SAS, France) containing plant extracts and seaweed, has been developed (28) and has been reported to improve growth performance in chickens in Germany and Egypt (29). The objective of the present study was, therefore, to determine the effect of the microencapsulated phyto/phycogenic NUQO©NEX supplementation on broiler growth performance, processing yields, meat quality, and sensory profile under the U.S. experimental conditions.

## Materials and methods

### Animal care

All animal experiments were approved by the University of Arkansas Institutional Animal Care and Use Committee (IACUC # 21050) and were in accordance with the recommendations in NIH’s *Guide for the Care and Use of Laboratory Animals*.

### Experimental diets and animal husbandry

A total of 1,197 male Cobb 500 broiler chicks were obtained from a commercial hatchery (Cobb Vantress, Siloam Spring, Arkansas) and transported to the University of Arkansas broiler research farm. Birds were fed a 3-phase feeding intervals consisting of a crumbled starter (1–14 day), pelleted grower (14–28 day), and pelleted finisher (28–40 day). Birds were randomly allocated into 57 total pens (21 birds/pen, 0.09 m^2^/bird) and assigned to one of three experimental treatments (Table 1): corn/soybean meal basal diet (CON), basal diet supplemented with NUQO©NEX (NUQO SAS, Annecy, France) phytogenic/phycogenic blend at 100 g/ton from d 1–28 then 75 g/ton from d 29–40 (NEX75), and basal diet supplemented with NUQO©NEX blend at 100 g/ton from d 1–40 (NEX100). The composition of these phytogenic/phycogeni additives are proprietary to NUQO SAS (Annecy, France), but they are polyherbal formulations of pre-standardized and tested herbs (encapsulated micro-particles of dried Kelp, Cinnamaldehyde, Thymol, and Eugenol). Diets were formulated to meet or exceed all nutrient recommendations from the commercial breeder standards (31), and were offered *ad libitum*. The doses were recommended by the manufacturer. Each pen consisted of litter top-dressed with pine shavings and was equipped with a nipple drinking system and bell feeder. A supplemental paper feed tray was used for the first 7 days. House temperatures were 32 °C at d1 and gradually decreased to 20 °C at d27. The photoperiod used was 24 L:0D (30 lux) on d 1, 23 L:1D (30 to 10 lux) from d 1 to d 10, and 18 L:6D (10 to 5 lux) for the remainder of the trial.

**Table 1:** Basal experimental diets.

Ingredient, % as-is	Starter (1–14d)	Grower (15–28d)	Finisher (29–40d)
Corn	51.6	56.85	61.85
Soybean meal	40.6	35.25	30.31
Poultry fat	3.91	4.26	4.59
Limestone	1.01	1.04	0.86
Dicalcium phosphate	1.49	1.32	1.26
Salt	0.41	0.41	0.41
Choline chloride	0.05	0.05	
L-Lysine HCl	0.22	0.21	0.20
DL-Methionine	0.31	0.26	0.21
L-Threonine	0.14	0.12	0.10
L-Valine	0.11	0.09	0.07
Sand	0.02	0.02	0.02
Minerals^a^	0.10	0.10	0.10
Vitamins^b^	0.025	0.025	0.025
**Calculated content, % unless noted otherwise** ^c^			
CP	23.3	21.1	19.1
ME, kcal/kg^d^	3,050	3,125	3,200
Ca	0.90	0.85	0.75
Na	0.18	0.18	0.18
DEB, mEq/kg^e^	257.7	231.4	208.1
Available P	0.42	0.38	0.36
Digestible, Lys	1.25	1.12	1.00
Digestible, Met	0.67	0.59	0.52
Digestible, TSAA	0.94	0.84	0.75
Digestible, Thr	0.85	0.76	0.68
Digestible, Val	0.96	0.86	0.77
Digestible, Arg	1.31	1.18	1.05

a The mineral premix contributed (per kg of diet): manganese, 100 mg; zinc, 100 mg; calcium, 69 mg; copper, 15 mg; iron, 15 mg; iodide, 1.2 mg; selenium, 0.25 mg.

b The vitamin premix contributed (per kg of diet): vitamin A, 7,716 IU; vitamin D3, 5,512 ICU; vitamin E, 55 IU (49.6 mg); niacin, 38.58 mg; d-pantothenic acid, 9.92 mg; riboflavin, 6.61 mg; pyridoxine, 2.76 mg; thiamine, 1.54 mg; menadione, 1.5 mg; folic acid, 0.88 mg; biotin, 0.08 mg; vitamin B12, 0.01 mg.

c Arg, arginine; Ca, calcium; CP, crude protein; DEB, dietary electrolyte balance; Lys, lysine; ME, metabolizable energy; Met, methionine; Na, sodium; P, phosphorus; Thr, threonine; TSAA, total sulfur amino acids; val, valine.

d EM was calculated using the equation given by Lodhi et al. (30).

e DEB (mEQ/kg) = [(Na^+^, g/kg/AMU) × 100] + [(k^+^, g/kg/AMU) × 100] – [(Cl^−^, g/kg/AMU) × 100].

### Growth performance, and processing parameters

Birds were weighed by pen at 1, 14, 28, and 40 day-post hatch and feed intake was recorded. Average body weight (BW), body weight gain (BWG), feed intake (FI), and feed conversion ratio (FCR) were calculated for starter, grower, finisher, and overall growth phase. Mortality and dead bird weights were recorded daily and then used to correct FCR, BWG, and FI per bird.

Feed was removed from all pens 10 h prior to processing, while water access was maintained. On day 40, 399 birds (7 birds/pen) were processed at the University of Arkansas Pilot Processing Plant (Fayetteville, AR) via a commercial inline system. Birds were weighed, placed on shackles, and electrically stunned (11 V, 11 mA for 11 s) before being exsanguinated, soft scalded (55°C for 2 min), de-feathered (Foodcraft Model 3; Baker international, MI, United States), and mechanically eviscerated. Carcass or weight without giblets (WOG), and abdominal fat pad weights were recorded immediately post evisceration. Carcasses were hot-deboned, following a brief ice bath, on a section of inline commercial deboning equipment (line speed set to 4″ of run time per second) with birds manually added to the initial cone. Attempting to reduce variability between part yield, trained individuals made identical cuts to debone whole carcasses into subsequent parts of skinless breast, tender, skin-on wing, and skin-on whole leg. Weights were then recorded and used to calculate yields relative to individual live bird weight.

### Blood chemistry, gas, and electrolyte parameters

On day 40, approximately 1 mL of whole blood was collected from brachial wing vein (cutaneous ulnar vein) using a 3 mL syringe and a 1-inch 20G needle and placed into K2 EDTA blood collection tubes (Becton Dickinson, Franklin Lakes, NJ) to prevent coagulation. pH, partial pressure of CO_2_ (pCO_2_), total CO_2_ (TCO_2_), partial pressure of O_2_ (pO2), bicarbonate (HCO − _3_), base excess (BE), O_2_ saturation (sO_2_), sodium (Na), potassium (K), ionized calcium (iCa), glucose, hematocrit (Hct), and hemoglobin (HB) analyses were determined via an i-STAT Alinity system (SN:801128; software version JAMS 88.A.1/CLEW D44; Abaxis, Union City, CA, United States) with the i-STAT CG8 + cartridge test (ABBT-03P88-25) according to manufacturer’s recommendation. Analysis was performed at room temperature using the temperature correction function of the i-STAT Alinity system. The i-STAT system has been validated in avian species (32–35).

### Femur- and tibia-lesion scoring

On day 40, 114 birds (2 birds/pen, *n* = 19/treatment) were humanely euthanized and immediately necropsied to determine presence of subclinical lesions in the proximal heads of both the femora and tibiae. Bone was selected macroscopically based on a previously reported scale (36, 37). Briefly, femur scores were classified into the following categories: 0- no sign of lesion and in-tact articular cartilage cap (Normal); 1- separation of the head from the acetabulum, called femur head separation; 2- necrotic lesion encompassing an area larger than approximately 1 cm^2^ (considered femur head necrosis or bacterial chondronecrosis with osteomyelitis). Tibial lesion severity was also scored on a 0 to 2 scale into the following categories: 0- no abnormalities (Normal); 1- transitional tibial head necrosis, 2- severe tibial head necrosis, characterized by degradation of internal growth plate structure due to infection.

### Woody breast and white striping scoring

Boneless breast filets were blind analyzed and macroscopically scored by a well-trained person (who did not know the treatments) for woody breast (WB) and white striping (WS) as previously described (38, 39). Briefly, for WB, a whole number increment scale was used with 0 being flexible throughout (normal), 1 being hard mainly in the cranial region (mild), 2 being mostly hard but some flexibility (moderate), and 3 being extremely hard throughout filet (severe). White striping was scored using 0 (normal), 1 (mild), 2 (moderate), and 3 having striations greater than 3 mm (severe).

### 24-h pH and color

Breasts filets were collected, placed on trays, covered with plastic overlay, and stored at 4°C for 24 h post-mortem drip loss, pH, and colorimetric analysis. Drip loss was calculated as the difference between hot debone breast weights and chilled breast weights and is expressed as a percentage of hot debone breast weight. Three readings were taken with a colorimeter (CR-400; Konica Minolta Sensing Inc., Sakai Osaka, Japan; size 102(W) X 217 (H) X 63 (D) mm) using illuminant D65 and a 2-degree observer to determine the L* (Lightness), a* (redness), and b* (yellowness) values on the ventral side of the left breast filet. Each left breast lobe was measured 3 times with a temperature-compensating pH meter (Testo 205; Testo Inc., West Chester, PA) to present an averaged breast filet pH.

### Sensory analysis and thiobarbituric acid reactive substances assay

At 47 days of age, following a 10 h feed withdrawal, 36 birds (12 birds/treatment) were randomly selected and transported to a commercial processing plant (B&R meat processing, Winslow, AR) to be slaughtered under federal (U.S. Department of Agriculture, Food safety and inspection service) inspection for subsequent sensory and TBARS analyses. After 24 h of air chilling at 4°C, the left breast filet was collected and delivered to the Sensory Science Center at the University of Arkansas Food Science Department for a professional meat descriptive sensory panel (IRB# 13-05-713). The descriptive panel was trained according to the Spectrum method (40) and has extensive experience with poultry and other meat products (41). Each panelist evaluated every sample at random for aroma, flavor, taste, and texture. All sensory attributes and texture characteristics were scored to the nearest 0.5 on a scale ranging from 0 (least intense) to 15 (most intense). TBARS were measured as previously describe (42, 43) with commercially available kits, which were utilized according to manufacturer’s protocol (Cayman Chemical Company, Ann Arbor, MI). Samples were analyzed at 1, 4, and 7 d postmortem and results are presented as mmol of malonaldehyde (MDA)/L.

### Statistical analysis

All data were analyzed using JMP Pro22 fit-model (SAS Institute, 2022, Cary, NC) within a randomized complete block design. Pen (*n* = 19/treatment) represented the experimental unit for growth performance, processing parameters, meat quality, bone lesion scores, and blood parameters. Bird (*n* = 12/treatment) represented the experimental unit for sensory and TBARS analyses. Outliers were removed from whole data set when multiple parameters exceeded ±2 SD. Data were analyzed with one-way ANOVA and Tukey as a multiple comparison test. Significance was set at *p* ≤ 0.05. In the ANOVA of the sensory data, panelist nested within session was included as a random effect in the model. Least square means were computed and separated with the pairwise *t*-tests (PDIFF option of SAS) when a significant (*p* < 0.05) *F*-test was noted.

## Results

### Dietary supplementation of NUQO©NEX improves growth performances in broilers

The growth performance, feed efficiency, and mortality percent are presented in Table 2. There were no differences observed in the 1–14d starter phase (*p* > 0.05) for any parameter between treatments. During the grower phase (14–28d), the NEX75 treatment had lower body weight gain (1.297 vs. 1.326 kg, *p* < 0.001), and feed intake (1.888 vs. 1.923 kg, *p* < 0.001) compared to the CON group (Table 2). During the finisher phase, the NEX100 treatment had higher body weight gain (1.838 kg vs. 1.776 kg, *p* = 0.0282) and averaged 3.7 points better FCR (*p* = 0.0528) compared to the CON group.

**Table 2:** Effect of different levels of NUQO© NEX on growth performance and mortality rate of male Cobb 500 chicks during different intervals of ages.

Treatment	BW^1^, Kg	BWG^2^, Kg	FI^3^, Kg	FCR^4^, g:g	Mortality, %
**Starter 1–14d**					
CON	0.630	0.584	0.604	1.072	0.53
NEX75	0.633	0.587	0.606	1.068	0.75
NEX100	0.631	0.584	0.601	1.071	1.34
SEM	0.003	0.003	0.003	0.005	0.459
*p*-value	0.8680	0.8485	0.6377	0.8109	0.5443
**Grower 15–28d**					
CON	1.956	1.326^**a**^	1.923^**a**^	1.451	1.06
NEX75	1.930	1.297^**b**^	1.888^**b**^	1.456	0.50
NEX100	1.952	1.321^**ab**^	1.908^**ab**^	1.445	1.08
SEM	0.009	0.006	0.008	0.006	0.442
*p*-value	0.0523	**0.0028**	**0.0131**	0.5295	0.6181
**Finisher 29–40d**					
CON	3.148	1.192^**b**^	2.104	1.771	2.65
NEX75	3.161	1.231^**ab**^	2.140	1.743	1.73
NEX100	3.206	1.252^**a**^	2.130	1.705	2.41
SEM	0.016	0.019	0.020	0.024	0.792
*P*-value	0.0976	**0.0300**	0.0957	0.0631	0.7611
**Period 1–40d**					
CON	3.148	3.102	4.631	1.513^**a**^	4.23
NEX75	3.161	3.115	4.634	1.506^**ab**^	2.98
NEX100	3.206	3.159	4.639	1.492^**b**^	4.83
SEM	0.016	0.016	0.017	0.005	1.076
*P*-value	0.0976	0.0967	0.9464	**0.0240**	0.5372

1 BW = 14d average individual pen body weight.

2 BWG = average individual body weight gain.

3 FI = average individual feed intake.

4 FCR = mortality corrected feed conversion ratio.

Different letters indicate significant differences at *p* < 0.05. Tukey–Kramer HSD test was used when appropriate. Bold value means *p* < 0.05.

When considering the whole growth phase performance, feed efficiency, and mortality rate, the NEX100 treatment averaged overall 2.1 points better FCR (*p* = 0.0240) and higher BWG (57 g, *p* = 0.0967) compared to the CON group. The mortality rate was not impacted by any of the experimental treatment.

As shown in Table 3, NEX supplementation did not affect blood chemistry, gas, and electrolytes.

**Table 3:** Blood gasses and electrolytes in 40d Cobb500 male broilers.

Treatment	pH	PCO_2_^1^	PO_2_^2^	HCO_3_^3^	BE^4^	sO_2_^5^	TCO_2_^6^	Na^7^	K^8^	iCa^9^	Glu^10^	Hct^11^	Hb^12^
CON	7.43	41.3	41.5	28.3	4.0	77.6	29.4	145.8	5.1	1.41	231.3	20.9	7.1
NEX75	7.44	40.7	42.8	27.4	3.2	79.3	28.6	146.4	5.0	1.39	229.5	19.8	6.7
NEX100	7.45	41.6	40.7	28.4	4.3	77.0	29.6	145.7	5.0	1.41	228.5	20.5	7.0
SEM	0.007	1.169	0.573	0.438	0.436	0.795	0.466	0.340	0.055	0.012	2.919	0.454	0.153
P-value	0.2218	0.9002	0.0591	0.3055	0.2927	0.1277	0.3654	0.2748	0.2553	0.4146	0.7327	0.2121	0.2019

1 PCO_2_ – partial pressure of carbon dioxide.

2 PO_2_ – partial pressure of oxygen.

3 HCO_3_ – bicarbonate.

4 BE, base excess, extracellular fluid compartment.

5 sO_2_ – oxygen saturation.

6 TCO_2_ – total carbon dioxide.

7 Na – sodium.

8 K – potassium.

9 iCa – ionized calcium.

10 Glu – glucose.

11 Hct – hematocrit.

12 Hb – hemoglobin.

### Effects of NUQO©NEX on the incidence of metabolic (bone and muscle) disorders

Tibia and femur lesion scores are presented in Table 4. The NEX100 treatment reduces the incidence of severe bacterial chondronecrosis with osteomyelitis (BCO, score 2) compared to the CON group (5.6 vs. 8.3%), however the differences were not statistically discernable at *p* < 0.05.

**Table 4:** Tibia and femur scores^a^ of 40d Cobb500 male broilers.

			Incidence of scores^b^, %		
Treatment	Left, Avg.	Right, Avg.	0	1	2
**Tibia**					
CON	0.417	0.528	61.1	30.6	8.3
NEX75	0.395	0.605	60.5	28.9	10.5
NEX100	0.222	0.528	68.1	26.4	5.6
SEM	0.103	0.121	6.363	5.766	3.085
*p*-value	0.3767	0.8118	0.5367	0.7406	0.5445
**Femur**					
CON	0.306	0.250	81.9	8.3	12.5
NEX75	0.316	0.211	78.9	15.8	5.3
NEX100	0.389	0.194	77.8	15.3	6.9
SEM	0.098	0.082	5.399	3.953	3.748
P-value	0.7026	0.9624	0.7028	0.2371	0.4152

a Scored on a numeric scale from 0 to 2: 0 = normal; 1 = tibia/femur head separation; and 2 = tibia/femur head necrosis.

b Scores were calculated on a per pen basis. Both tibias/femurs for 2 birds were used to calculate pen Incidences.

As shown in Table 4, although it is not statistically significant, both NEX (75 and 100) treatments reduced the incidence of severe BCO (score 2) (12.5% vs. 5.3 and 6.9% for CON, NEX75, and NEX100, respectively), but increased that of score 1 compared to the CON group (8.3% vs. 15.8 and 15.3% for CON, NEX75, and NEX100, respectively).

Both NEX treatments increased total incidence of muscle (WB and WS) myopathies, although the differences were not statistically discernable at *p* < 0.05. Birds fed NEX75 had a higher score for all WB scores, while birds fed NEX100 had lower WB score 2, but an increased WB score 1 and 3 compared to the CON group (Table 5). Furthermore, birds fed NEX75 had a lower WS score 2 compared to the CON, while birds fed NEX100 had a lower WS score 1, but an increased score 3 (Table 5).

**Table 5:** Woody breast and white striping score^a^ of 40d Cobb500 male broilers.

	Incidences (%)				
Treatment	0	1	2	3	Total^b^
**Woody breast**					
CON	60.2	24.7	14.2	0.8	39.8
NEX75	49.4	28.1	18.2	4.5	50.6
NEX100	52.4	30.2	11.9	5.4	47.6
SEM	4.300	3.876	2.496	1.511	4.300
*p*-value	0.2705	0.6373	0.1845	0.1830	0.2705
**White stripe**					
CON	40.4	40.4	18.3	0.8	59.6
NEX75	39.5	43.2	15.3	2.3	60.5
NEX100	38.4	38.7	18.2	4.7	61.6
SEM	4.448	4.473	3.087	1.317	4.488
P-value	0.9864	0.6785	0.7452	0.1199	0.9864

a Scored on a numeric scale from 0 to 3: 0 = no woody breast; 1 = mild woody breast; 2 = moderate woody breast; 3 = severe woody breast.

b Any incidence of woody breast.

### Effects of NUQO©NEX on processing-, meat quality-, and sensory-parameters

Processing part weights are presented in Table 6. The NEX100 treatment had heavier body weight (3,206 kg vs. 3,124 kg, *p* = 0.028) and carcass weight (2,361 kg vs. 2,292 kg, *p* = 0.014) compared to the CON group. The NEX100 treatment also had larger breast (656 g vs. 626 g, *p* = 0.0570) and heavier leg quarters (730 g vs. 714 g, *p* = 0.05) than the CON group.

**Table 6:** Processing weights of 40d Cobb500 male broilers.

Treatment	BW^1^, g	WOG^2^, g	Fat^3^, g	Breast^4^, g	Tender^5^, g	Wings^6^, g	LQ^7^, g
CON	3,124^b^	2,292^b^	19	626	115	244	712
NEX75	3,145^ab^	2,310^ab^	19	643	116	245	714
NEX100	3,206^a^	2,361^a^	19	656	118	249	730
SEM	20.135	15.878	0.718	7.870	1.150	1.746	5.652
P-value	***0.0282***	***0.0141***	0.8396	0.0570	0.2992	0.2097	0.0503

1 BW = 40d dock body weight.

2 WOG = carcass weight without giblets.

3 Fat = hot fat pad.

4 Breast = skinless Pectoralis major.

5 Tender = Pectoralis minor.

6 Wings = skin-on wings.

7 LQ = skin-on drum and thigh; different superscript letters indicate significance difference at *p* < 0.05. Bold value means *p* < 0.05.

Meat quality data are presented in Table 7. The NEX75 treatment breast filets had more yellowness than the CON and NEX100 treatments (*p* = 0.003). There were no differences observed in 24-h post-mortem breast filet pH, drip loss, lightness, or redness (*p* > 0.05).

**Table 7:** Meat quality of 40d Cobb500 male broilers^1^.

			Breast filet colour^2^		
Treatment	pH	Driploss, %	L*	a*	b*
CON	5.87	2.07	54.80	4.71	10.27^b^
NEX75	5.88	2.51	55.18	4.64	10.94^a^
NEX100	5.89	2.36	54.81	4.68	10.23^b^
SEM	0.012	0.163	0.216	0.109	0.149
P-value	0.3793	0.1787	0.3584	0.8156	***0.0026***

1 Parameters were measured 24-h post-harvest.

2 L* - lightness; a* - redness; b* - yellowness.

*p*-value < 0.05 were considered significant; Tukey HSD was used when appropriatedifferent superscript letters indicate significance difference at *p* < 0.05. Bold value means *p* < 0.05.

**Figure 1:**
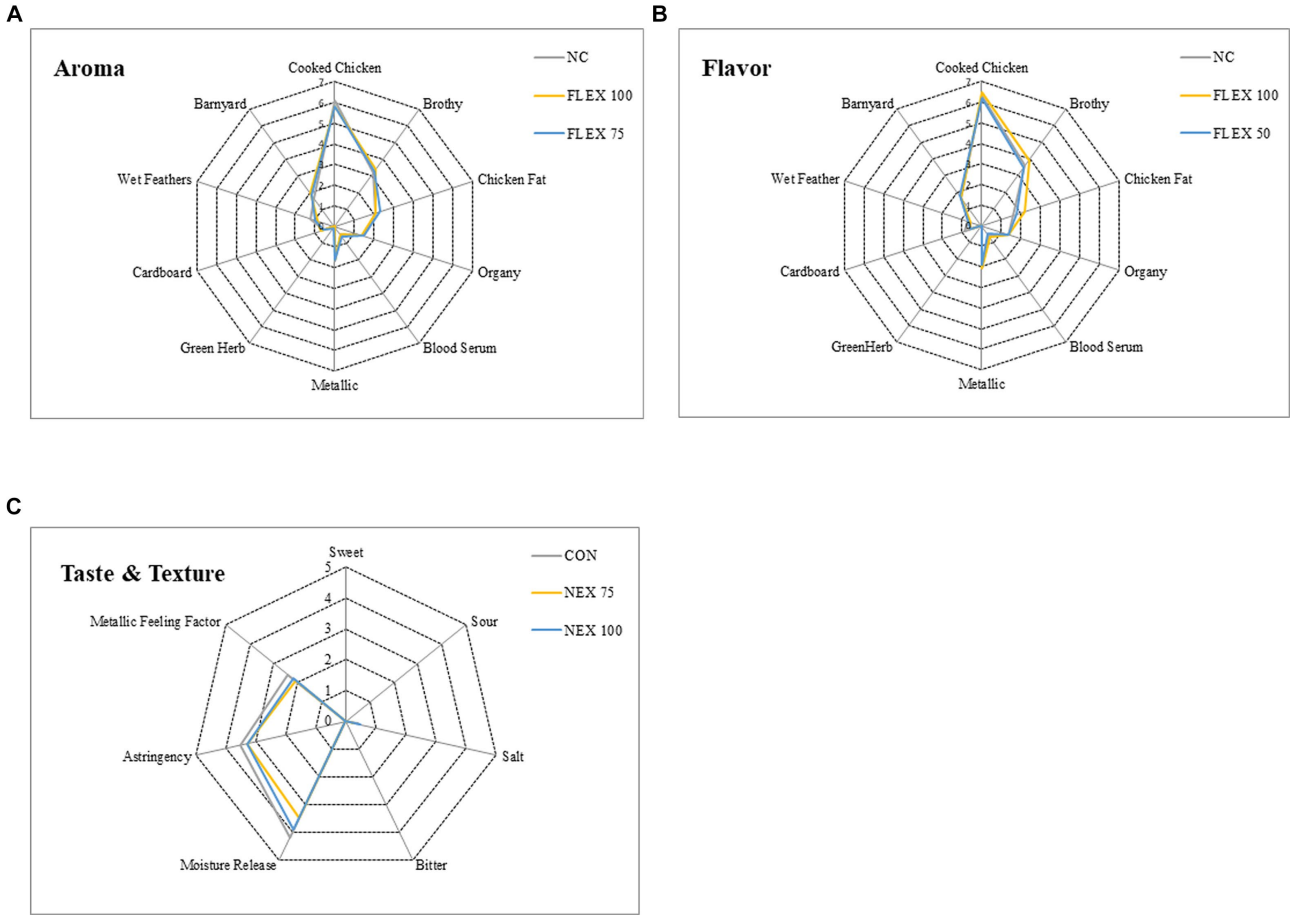
Effects of NUQO©NEX supplementation on broiler breast meat sensory profile. After 24 h of air chilling at 4°C, the left breast filet was collected and delivered to the Sensory Science Center at the University of Arkansas Food Science Department for a professional meat descriptive sensory panel. Each panelist evaluated every sample at random for aroma **(A)**, flavor **(B)**, and taste and texture **(C)**. All sensory attributes and texture characteristics were scored to the nearest 0.5 on a scale ranging from 0 (least intense) to 15 (most intense).

Sensory profile data are presented in Figure 1. There were no differences in any aroma (Figure 1A), flavor (Figure 1B), or taste/texture (Figure 1C) of the breast filets (*p* > 0.05).

TBARS data are presented in Table 8. Although the differences were not statistically discernable, NEX100 treatment reduced the levels of breast filet TBARS at 1, 4, or 7 days-post harvest (*p* = 0.5663, *p* = 0.3150, and *p* = 0.053, respectively).

**Table 8:** Breast filet TBARs of 40d Cobb500 male broilers.

	Concentration, mmol/L			
Treatment	Day 1	Day 4	Day 7	Mean
CON	26.4	32.1	19.3	25.9
NEX75	26.7	32.7	36.0	31.8
NEX100	20.8	21.0	17.8	19.9
SEM	4.375	6.015	5.650	3.381
*p*-value	0.5663	0.3150	0.0533	0.0574

## Discussion

With an anticipated annual market growth of 7.2% from 2022 to 2027, blended phytogenic feed additives have tremendous potential to promote health, performance, and uniformity in animal production (44). Overall, performance results of phytogenic inclusion in poultry diet have been inconsistent, which is accredited to the wide range of compositions of the active ingredients being used (6). The exact underlying mechanism for every phytogenic is complex and remains to be fully elucidated, yet recent emerging scientific findings are starting to provide a better mechanistic understanding of the mode of action of these natural feed additives.

The composition of the new microencapsulated NUQO©NEX feed additive is a proprietary of NUQO SAS (Annecy, France), but is a polyherbal formulation consisted of a combination of metabolites from plants and from marine algae (phytogenic and phycogenic). It contains dried kelp and the essential oils thymol, cinnamaldehyde and eugenol (derived from thyme, cinnamon and cloves, respectively) encapsulated in a fat matrix. Correctly identified, validated, and blended phytogenics can exhibit synergistic effects that can enhance their antimicrobial and antioxidant bioactivity, while being included at much lower concentrations (20). Phytogenics also need to be stabilized to survive processing conditions and delivered at the right place in the digestive tract to exert their activity in the animal (45).

In our experimental conditions, all treatments exceeded breeder’s growth guide (31). This optimization of broiler genetic potential was the result of optimal environmental conditions, adequate nutrient supply, and the absence of any extrinsic health challenges, which was supported by the lack of any significant differences in blood chemistry, gas, and electrolytes, as well as bone attrition. It is worth noting that although the differences were not statistically significant, the supplementation of NUQO©NEX reduce the severity of both tibia and femur BCO, which merits further investigations.

Of particular interest, the in-feed supplementation of microencapsulated NUQO©NEX at higher dose, resulted in an average 57 g greater BWG, 2.1 better FCR, and 30 g heavier breast weight compared to their counterpart control birds that were already at or close to genetic potential. The rule of thumb, as the 2021-global broiler production was ~71 billion birds, is that NUQO©NEX supplementation would increase the worldwide broiler meat production by 4 million tones, with a 2.1 million tones improvement in breast weight. The observed improvement here in growth performance was not surprising as it has been reported that NUQO©NEX ameliorate nutrient and mineral digestibility and thereby enhance performance at 21 days with better gain (+2.2%) and better FCR (−0.6%) (29). Furthermore, phytogenic combinations such as thymol, cinnamaldehyde, eugenol, and various others have had positive impacts through increased BWG and decreased FCR (46–52). Bravo and Ionescu concluded in a 13 trial meta-analysis that blended products (carvacrol, cinnamaldehyde and capsicum oleoresin) increased BWG, while reducing FCR and mortality (53). Contrarily, Najafi and Torki found that only thymol positively impacted BWG and FCR, while cinnamaldehyde and eugenol showed no effects on performance (54). In another 124 trial meta-analysis, it was concluded that a blend of thymol, carvacrol, and cinnamaldehyde displayed a higher inclination for improved performance and feed efficiency (55). The differences observed in the beneficial effects of the aforesaid phytogenics might due to their compositions, dosage, intestinal stability of their active compounds, chicken strains, diet composition, and/or experimental conditions and duration.

Although it was not studied in combination with phytogenics, increasing number of investigations have shown that dietary administration of algae alone improved also growth performances in poultry (2, 25, 56–58). Here, it is clear that the encapsulated phytogenic-phycogenic combination synergistically improved growth performance in broilers, and therefore its underlying mechanism warrants further in-depth investigations.

Although its mode of action warrants future studies, it is possible that the microencapsulated NUQO©NEX has a good protection of its active ingredients, a better stability, and an optimal release in the gut. This, in turn, would improve macronutrient and mineral digestibility and boost broiler performances. It is also possible that NUQO©NEX exerts a cytoprotective effect via its antioxidant, immunomodulatory, and/or anti-inflammatory property (28). Furthermore, Dridi’s group has recently shown that in feed- or in water-phytogenics administration improved growth performances via modulation of feeding-related hypothalamic neuropeptides and peripheral intermediary (lipogenic and lipolytic program) metabolisms (59–62). According to recent reports, there is increasing evidence that phytogenics stabilize and improve gut microbiota, which in turn enhance intestinal integrity and health leading to better growth (63–66). Although these speculations for NUQO©NEX’s mode of action are scientifically sound, they need to be supported and proven by mechanism-based studies.

Regarding processing weights and meat yield, in contrast to CON birds that lost an average of 24 g/bird after 10 h feed withdraw, birds fed NUQO©NEX at higher dose maintained the same body weight. This could be a result of a better gut integrity and health and improved resilience to the nutritional withdraw stress. Furthermore, NUQO©NEX birds had higher numerical breast meat yield (+0.42%) compared to the CON group, which could have a significant economic impact on the industry, especially in regions such as the U.S.A. where more than 90% of the consumers strongly prefer breast meat to the alternative dark meat (67). Although the effect was not statistically significant, the flip side of the coin is that this improvement tended to increase the incidence of muscle (WB and WS) myopathies. This is not surprising as body weight has shown to have a direct positive correlation with incidence of severity of WB and WS (68). The comparison to the current control is, however, unfair as it has a lower body weight. Essentially, the effect of NUQO© NEX on muscle myopathies should be studied versus a control at iso-weight.

The breast filet quality (pH, drip loss, and color) from the current trial are in close alignment with other experiments conducted with the same equipment (69). The postmortem pH can have large impacts on meat quality through altered functionality, color, drip loss, and shelf life (70). The observed values (~5.8) here are considered in the “normal” range of 5.7–6.1 (71–73). The breast filets from the NEX75 treatment had more yellowness than both other treatments. Broiler meat color can be impacted by a variety of factors including sarcoplasmic protein (myoglobin, hemoglobin, cytochrome, catalases, etc.), pH, age, sex, breed, management, processing techniques, and/or diet (67, 74). Phytogenic feed additives have displayed tremendous potential to improve pigmentation. A study by Reis et al. found that breast filet yellowness increased when broilers were fed a phytogenic blend of carvacrol, thymol, and cinnamic aldehyde (75). Similar results were observed from another group, which found that thymol, cinnamaldehyde and carvacrol increased yellowness with and without curcumin (76). On the other hand, Dridi’s group and Alfaia and co-workers have shown that algae treatment increased meat yellowness (b*), which is probably due to high levels of carotenoids and retinol (77).

## Conclusion

In conclusion, although meat quality was not altered, the supplementation of the phyto/phycogenic NUQO©NEX improved finisher performance parameters, whole phase FCR, processing carcass weights, and breast filet yellowness, at varying inclusion levels. Further titration and mechanistic studies are warranted to identify the most beneficial dose of NUQO©NEX and to define its mode action.

## Data availability statement

The original contributions presented in the study are included in the article/supplementary material, further inquiries can be directed to the corresponding author.

## Ethics statement

All animal experiments were approved by the University of Arkansas Institutional Animal Care and Use Committee (IACUC # 21050) and were in accordance with the recommendations in NIH’s Guide for the Care and Use of Laboratory Animals. The study was conducted in accordance with the local legislation and institutional requirements.

## Author contributions

GM: Data curation, Formal analysis, Writing – original draft. EG: Data curation, Writing – review & editing. AR: Data curation, Writing – original draft. CM: Data curation, Formal analysis, Writing – original draft. SD: Conceptualization, Funding acquisition, Visualization, Writing – original draft, Writing – review & editing.
